# Clinical investigation for displaced proximal humeral fractures in the elderly: a randomized study of two surgical treatments: reverse total prosthetic replacement versus angular stable plate Philos (The DELPHI-trial)

**DOI:** 10.1186/1471-2474-15-323

**Published:** 2014-09-28

**Authors:** Tore Fjalestad, Petter Iversen, Margrethe Øye Hole, Morten Smedsrud, Jan Erik Madsen

**Affiliations:** Orthopaedic Department, Division of surgery and Clinical Neuroscience, Oslo University Hospital HF, Nydalen, Postbox 4950, Oslo Norway; Department of Physiotherapy, Division of Medicine, Oslo University Hospital HF, Oslo, Norway; Orhopaedic Department, Asker and Bærum Hospital, Vestre Viken HF, Bærum, Norway

**Keywords:** Proximal humeral fracture, Surgical treatment, Randomized clinical trial, Reversed shoulder prosthesis, ORIF, Philos plate, Physical therapy, Semi-blinded

## Abstract

**Background:**

Treatment for displaced proximal humeral fractures is still under debate. Few studies exist at the highest level of evidence. Although reversed total shoulder prosthesis has gained popularity and showed promising results in the treatment for proximal humeral fractures in the elderly patients, no randomized controlled trials exist to the authors’ knowledge.

**Methods/Design:**

This study is a randomized semi-blinded controlled multicenter trial designed according to the Consort statement and the recommendations given by the Cochrane reviewers for proximal humeral fractures. The study will investigate whether a reversed total shoulder prosthetic replacement gain better functional outcome compared to open reduction and internal fixation using an angular stable plate in displaced three- and four parts proximal humeral fractures after two and five years follow-up.

Participants are aged 65–85 admitted in seven different hospitals with a displaced proximal humeral fracture according to AO-OTA type 11-B2 or 11-C2. The intervention group is surgical treatment using a reversed total shoulder prosthesis (Delta X-tend) compared to open reduction and internal fixation with an angular stable plate (Philos) and thread cerclage in the control group. 60 patients will be randomized to each group.

The primary outcome is shoulder function (Constant score). Secondary outcomes will be patient self-assessment form (Oxford shoulder score), a quality of life questionnaire (15D score) and resource implications (cost-effectiveness). Follow-ups take place at 3, 6, 12 and 24 months, and five years. The trial design is semi-blinded with blinded physiotherapists performing the functional testing of patients at all follow-ups.

Randomization to treatment groups is electronic online, by independent supervisor (web-CRF). The recruitment of patients started at January 1.st 2013. Inclusion of 120 patients during three years is expected.

**Discussion:**

This semiblinded trial include a high number of patients compared to existing randomized trials in this field. To our knowledge and according to ClinicalTrials.gov, this is the first study that compare these two treatments for a displaced proximal humeral fracture in elderly patients. This may provide important information to help the surgeon to decide the best treatment in the future.

**Trial registration number:**

ClinicalTrials.gov Identifier: NCT01737060

**Electronic supplementary material:**

The online version of this article (doi:10.1186/1471-2474-15-323) contains supplementary material, which is available to authorized users.

## Background

Treatment for displaced proximal humeral fractures is still under debate several decades after the first recommendations for “modern treatment principles” were given by Neer [1, 2]. Meta-analyses have been difficult to perform due to different methodological approaches, treatment modalities and rehabilitation programs. According to the last Cochrane review, including 16 small randomized trials at the highest level of evidence and a total number of 801 patients, best treatment has not yet been ruled out. The authors of the Cochrane review emphasize that new trials must take into account important issues like methods of randomization, blinding and duration of follow-up, as well as standard and validated outcome measures, patient assessed functional outcomes and resource use assessments. New trials should also meet the criteria for design and reporting according to the Consort statement [3, 4].

Our intention is to design a randomized study between two methods of surgical treatment meeting the recommendations given by these authors.

Fractures of the proximal humerus normally account for about 5% of all fractures. Most patients sustaining these fractures are women above the age of 60. The incidence of the proximal humeral fracture has been reported to increase with age during several decades, also based on the age-adjusted incidence [5–8]. Ageing of the world population will clearly increase the number of future fractures, as high age, incidence of falls and osteoporosis are the main risk factors [9–12]. Fractures in the elderly represent in general a considerable burden to the patients in terms of pain, loss of function and even mortality, and to the society generating costs in different aspects [13]. Physical disabilities caused by fractures of the proximal humerus also make self-care and independent living difficult, thus interfering with quality of life [14]. In light of this, fractures in the elderly will become increasingly important in the future.

Although numerous studies exist dealing with proximal humeral fractures, most of them represent level IV evidence [15]. Thus, interpretation must be made with caution and evidence is at present too limited to support secure recommendations for treatment, even including if surgical treatment is superior to conservative for severely displaced fractures. The number of level I and II trials in this field are few. In our department we recently completed a single center randomized controlled study of displaced proximal humeral fractures; an angular stable plate was compared to conservative treatment. After two years follow-up we could not detect any significant differences between surgery and conservative treatment with regard to physical functioning and health-related quality of life [16, 17]. Nevertheless, there is evidence that the rate of surgical treatment has increased significantly in the period from 1999 to 2005 [18].

The proximal humeral fracture groups comprise two-, three,- or four part fractures: non-displaced, displaced or displaced with dislocation (AO-OTA type A,B and C) [19]. Many proximal humeral fractures are minimally displaced, and should not be considered for surgical treatment. About one fifth are displaced 3- and 4 part fractures (AO-OTA group B2 and C2) [7]. In these fractures many surgeons perform operative treatment, even if this has not been proven superior to a conservative approach except from in certain displaced fracture dislocations, where surgery may be favorable [3]. However, most orthopaedic surgeons agree that classification and differentiation of the main groups of proximal humeral fractures are important for treatment decision making, as prognosis and the technical challenges depend on the fracture displacement and comminution [20, 21].

If surgery is decided, the most frequent treatment modalities are open reduction and internal fixation with an angular stable plate, a locked intramedullary nail, or in some patients minimally open surgery with screws, threaded pins and sometimes cerclage wires. Shoulder hemi-artroplasty has also been widely used [22–32]. Although numerous surgical options exist for the displaced fractures, none has yet proven to be superior [3, 33–35]. Also, several complications and impaired shoulder function are reported after different types of surgical treatment [36–43].

The early reports of treatment with ORIF using angular stable plates were promising [44]. Later, however, it has become evident that although the angular stable implants contributed to the surgical management of severely displaced fractures, several problems and pitfalls stayed unsolved. The method of fixation utilizing an angular stable plate caused a disappointing number of complications, as cut-out of screws into the joint space, avascular humeral head necrosis, non-union, and varus malalignment [45–49]. Some of these complications are caused by poor surgical technique, and may be avoidable [50]. Supplemental tension band fixation and restoration of the medial calcar may improve outcome and decrease the incidence of hardware-related complications [20, 22, 51].

Head replacement or shoulder hemi-artroplasty (HA) has been used as a treatment option for three decades, and has been claimed a better option than open reduction and fracture fixation in certain severely displaced and dislocated intracapsular fractures, even though the evidence is still not conclusive. Some related important problems are well known: Poor rotator cuff status and non-union of the tubercles. If these main problems are avoided, patients may have a good outcome with these implants [41]. In the elderly patient suffering a proximal humeral fracture, however, the status of the rotator cuff frequently is poor [42, 43, 52, 53]. A recent randomized study concluded that head replacement did not prove better than conservative treatment [35], and several other authors also report unsatisfactory results with hemi-arthroplasty; Loss of ROM, non-union of tubercles in as many as 50% of the patients in some series and poor quality of the rotator cuff result in inferior functional outcome [28, 41, 54, 55].

A more recent treatment option for displaced proximal humeral fractures is the reverse total shoulder artroplasty (RTSA). It was introduced nearly 20 years ago in treatment of acute proximal humeral fractures [56]. During the last decennium the use of RTSAs has increased, both as a salvage procedure after failed hemi-artroplasty but also in the primary fracture care. RTSA has gained popularity and has showed promising results in the treatment for proximal humeral fractures in the elderly patients [57–59]. A small level 2 study was recently reported [39], concluding that RTSA showed similar outcome to HA, but functional outcome was more predictable. The advantage of the concept is less dependence on a normal rotator cuff, as the elderly population frequently suffer asymptomatic degenerative cuff tears leading to a poor prognosis [60]. However, problems with RTSA have been reported, mainly instability, poor rotation and fatigue of the deltoid muscle with time [56]. Also, radiological notching of the glenoid is frequent, but has not proven clinically important in the older Delta III prosthetic designs [61]. Due to these problems and complications the RTSA has until recently only been advocated in older patients [37]. Recently improved prosthetic designs possibly have reduced the problem of notching, due to overhang of the scapular component [62].

### Aims – hypothesis

#### Primary objective

The study will investigate whether a reversed total shoulder prosthetic replacement gain a better functional outcome compared to open reduction and internal fixation using an angular stable plate in severe displaced proximal humeral fractures.

We aim to conduct a high quality randomized controlled trial according to the recommendation from the Cochrane authors. Standard and validated outcome measures, patient assessed functional outcomes and resource implications like cost-effectiveness of these surgical methods will be addressed.

#### Secondary objective

Quality of life will be measured with the 15D score [63], and calculations of QALY and cost during hospital stay and two years follow-up. Further outcomes are radiographic results and the Oxford shoulder score [64].

#### Hypothesis

We aim to establish if treatment with reverse total shoulder artroplasty (RTSA) gain better functional outcome according to the Constant shoulder score compared to open reduction and internal fixation with an angular stable implant at two and five years of follow-up [65, 66].

## Methods/Design

### Study design

The design of the study is a multi-centre randomized controlled trial (RCT). Patients admitted to hospital with a displaced proximal humeral fracture in need of surgical treatment will be randomly allocated to two groups; Intervention group or control group. The design is semi-blinded for independent physiotherapists interviewing and testing the patients at three and six months, thereafter at one, two and five years.

### Interventions

**Group 1** is the intervention group. Patients will be treated surgically with a deltopectoral approach, resection of the supraspinatus tendon and insertion of a monobloc semented reversed total shoulder artroplasty of Delta Extend type (DePuy, Johnson & Johnson, UK).

**Group 2** is the control group. Patients will be treated surgically with open reduction and internal fixation using the Philos angular stable plate (Synthes, Switzerland) and thread cerclages to secure the tubercles and rotator cuff insertions to the plate. If necessary, judged by the surgeon, a bone substitute or bone graft will be used to enhance stability of the fracture (Norian® or autologous bone graft from the iliac crest).

For both groups the operations will be performed by experienced ortophaedic surgeons well trained within these procedures. In the postoperative treatment early exercises are emphasized in both groups, and will be guided by a specific training protocol recommended to outclinic physiotherapists (Table 1).

**Table 1:** **Timeline for physical therapy guideline**

Elements of physical therapy	Reversed prothesis	Philos
	Group 1	Group 2
Anti edema elbow, hand, fingers	Day 1	Day 1
Pendula exercises, passive assisted exercises in forward elevation, extension and abduction.	Day 1	Day 1
Active assisted exercises, Stability exercises and positioning of the scapula	Day 8	Day 5
Active assisted Internal + External Rotation	3 weeks	Day 5
Active exercises. Functional exercises	6 weeks	3 weeks
Isometric resistance	8 weeks	4 weeks
Active strengthening exercises	8 weeks	6 weeks
Functional strengthening exercises and proprioceptive exercises-restore dynamic stability.	12 weeks	8 weeks

### Method of inclusion

Patients will be recruited from six collaborating hospitals. An experienced senior consulting orthopaedic surgeon at the local department will classify the radiographs before electronically transmitting them to the study center (OUS Orthopaedic Department), where another consulting orthopedic surgeon experienced in skeletal traumatology will confirm radiographs and inclusion in the trial before randomization and allocation to treatment.

Allocation to treatment will take place after thorough oral and written information to the patient, and signed consent. Electronic randomization will be performed by a dedicated doctor at each hospital, and by means of a secured web-solution made by NTNU WebCRF system, with approval from the OUS Head of Patient Security (https://webcrf.medisin.ntnu.no/client/index.php).

### The inclusion criteria

Patients aged 65 or older admitted to hospital with a displaced three- or four part proximal humerus fracture of OTA/AO group 11-B2 or 11-C2 (displaced fracture of extra-articular or articular, bifocal type) [19]. The subgroups -.1, -.2 and -.3 will be included for both B2 and C2 groups, provided severe displacement, defined as a mal-position of at least 45° of angular deviation in valgus or 30° in varus in true frontal projection, regardless of whether the fracture is impacted or not. Fracture with more than 50% displacement of the head against the metaphysis (surgical neck) will also meet the criteria. The greater or lesser tubercles must be displaced in a three- or four part fracture, the degree of displacement is not critical for inclusion.

### Exclusion criteria

Patient younger than 65 years or older than 85 years. Previous history of injury or illness of the injured or contra-lateral shoulder, injuries of other parts of the humerus or the contra-lateral upper extremity, alcohol- or drug abuse, dementia, neurological diseases, or severe cardiovascular or lung diseases that would contraindicate surgery. Patients must understand the Norwegian language and be compliant to rehabilitation and follow-ups. A fracture or severe deformity of the glenoid should not appear in a pre-operative CT-scan of the shoulder.

### Ethics and safety

The study has been approved by the Regional Committee of Research, Health Region Southeast, Oslo, Norway at November 6^th^ 2012 (Reference 2012/1606).

Patients will be included after thorough oral and written information in accordance with regional ethic committee approval. All patients must give their written consent.

A study specific participant’s number and code referring to their hospitals will identify the participants. Patient name and other identifiable details will not be included in any electronic study data file. Any patient related data transferred between the main study group at OUS and participating sites will be identifiable only by the patients unique study number.

### Outcome parameters

Functional score of both shoulders, QoL interviews (15D) as well as health economic evaluation will be performed by an independent physiotherapist blinded to the treatment. Self-assessment score (Oxford shoulder score) will be included at each follow-up.

### Primary outcome: Constant score

The score originally described by Constant and Murley will be used to evaluate the functional outcome [65]. Maximum score for each shoulder is 100 points. Both shoulders will be rated and presented as absolute scores (CS). In order to reduce the influence of age the difference between the scores of the injured and the uninjured shoulder will be calculated. This difference is designated CSD (Constant Score Difference). Thus, normal function on the injured side will be zero. Measurement of strength will be performed according to recommendations given by the European Society of Shoulder and Elbow Surgeons ESSSE (http://secec.org/). The highest value of three measurements will be registered for the power score. There is no established minimal clinically important difference (MCID) for the Constant score, but 10 points is often regarded as suitable, although debated [67, 68].

The “Adjusted Constant score” (ACS) for the injured shoulder will also be calculated from the CS at the injured shoulder with adjustment for age and gender as a secondary outcome [66].

### The oxford shoulder score

The Oxford Shoulder score constitutes of twelve questions specific to shoulder function subdivided in five different response levels. The score is presented in the Norwegian translation in Additional file 1 by appointment with Isis Innovation Limited (Project number 3240) and professor A. Carr, University of Oxford, UK, who has contributed to the development of this test [69]. The score consists of twelve questions concerning shoulder function and activity of daily living, each question with five response levels. Scoring differ from 12 (worst) to 60 (best) points.

### Health related quality of life 15D

We use Harri Sintonen’s “15D”, a generic, self-administered instrument to assess Health Related Quality of Life (HRQoL) [63]. It encompasses 15 dimensions of health. The dimensions are moving, seeing, hearing, breathing, sleeping, eating, speech, eliminating, usual activities, mental function, feeling of discomfort, depression, distress, vitality and sexual activity. The questionnaire is modified, as the last dimension sexual activity is omitted, as some elderly patients may feel uncomfortable with this question. This score is validated and modifications in the statistical syntax for calculation of the score has been made to avoid confounding the outcome (http://www.15D-instrument.net). The scores along the 15 dimensions are translated into a zero-to-one quality of life index by means of an algorithm where zero represents death and 1.0 represents perfect health.

On admission to hospital, the patients will be asked to report their HRQoL as it was immediately before the fracture, and it will be repeated at 6 months, one, two and five years follow-up.

Quality adjusted life-years (QALYs) will be calculated for each patient by multiplying the time spent in a health-condition since the last follow-up by the HRQoL of this condition using the 15D scores (Additional file 1).

### Radiographic evaluation

Initial radiographic examination will be performed with plain X-ray projections. These are:

1. True antero-posterior projection.
2. Scapula-Y projection
3. Computer-tomographic scans including 3D scans will also be performed to secure optimal classification pre-operatively, as the problem of intra- and inter-observer reproducability is a well known confounder of fracture classification in proximal humeral fractures [70, 71].

The fracture patterns will be classified according to the OTA/AO-system into types, groups and subgroups [19].

The medial hinge is measured on the initial radiographic examination. This is an important predictor of avascular humeral head necrosis as well as failure of fixation when treated with ORIF, as it offers mechanical support and maintain perfusion of the humeral head by the vessels in the posteromedial periosteum [51].

### Scoring of radiographs

Rating of implant position for RTSA and Philos plate will be according to figures in Additional file 1.

Radiographic evaluation by two independent reviewers is an important principle when radiographs are scored. All radiographs from all centers will be examined by the same two independent senior consultants: One experienced skeletal radiolog and one orthopaedic surgeon who had not participated in the surgical treatment of these patients. Scoring of radiographs from three and six months, one, two and five years will be performed. All radiographic measurements will be performed using the Siemens Magic Web 300 software.

### Important items for ORIF with Philos plate

Pre-operative:

- Initial valgus or varus displacement (degrees°)
- Medial metaphyseal comminution, i.e. intermediate cortical fragment (Yes/No)
- Medial metaphyseal head extension in varus displacement (millimeters) [51]
- Medial hinge displacement in valgus displacement [51]

Post-operative and later follow-ups:

- Humeral head inclination
- Position of greater tubercle and plate height. Displacement, resorption or healing.
- Alignment of the humeral head in glenoid fossa (central or acentric).
- “Sinking” of humeral head resulting in late screw tip penetration of joint surface
- Avascular head necrosis according to our former classification (Grade 0-1-2) [16]*.
- Failure of osteofixation (pull-out, cut-out)
- Non-union

*Definition of avascular humeral head necrosis as seen on plain X-ray projection will be:

- 2 points: No changes
- 1 point: Changes in normal trabecular organization engaging less than 50% of the humeral head in true AP view
- 0 point: Partial collapse of the humeral head surface and-/or structural changes engaging more than 50% in true AP view.

### Important items for Reverse Total Shoulder Arthroplasty (RTSA)

Preoperative:

- Metaphyseal comminution (cerclage wire around the metaphysis necessary?)
- Glenoid anteversion or retroversion

Postoperative and later follow-ups:

- Notching will be evaluated according to Nerot score [72, 73]
  - Grade 1: notch limited to the scapular pillar
  - Grade 2: notch reaching the inferior screw of the base plate
  - Grade 3: notch extending beyond the inferior screw
  - Grade 4: notch reaching the base-plate’s central peg
- Position of greater and lesser tubercles. Displacement, resorbtion or healing
- Ossifications of the scapulohumeral space
- Radiolucent lines will be measured a) in seven different zones around the humeral stem, and b) beneath the glenoid baseplate and around the central peg and fixation screws [74] Additional file 1: Figure S3.
- Dislocation

### Economic evaluation

Cost data in this study will include costs incurred in hospital and subsequently, including time spent in operating theatre, hospital consumables, rehabilitation stays, subsequent hospital stay, help at home, consultations of NHS care (General practitioners, physiotherapists, district nurses etc.), transportation and sick-leave. Information for estimating costs will be collected from the patient by a questionnaire at each follow-up. Health-utility data will be obtained from the HS15D [63] (Additional file 1).

### Clinical follow-ups and blinding

The clinical follow-ups will take place at 3 and 6 months, one, two and five years. An independent physiotherapist will perform Constant scoring and assist the patients with a 15D QoL-questionnaire at these follow-ups. The physiotherapist will be blinded to the treatment and shall not take active part in the patient treatment of either group. The postoperative exercises and prescribed self-training program will be explained to patients by another physiotherapist before they are discharged from the hospital.

At every follow-up all patients will wear a T-shirt to cover their shoulders during examination by the physiotherapist performing the shoulder test. The patients will be thoroughly instructed not to tell the physiotherapist about their treatment.

Patients will complete the OSS and ASES self-assessment form at 6 months, one, two and five years follow-ups. Radiographic examination will be performed at every follow-up.

### Timeline - inclusion period

Inclusion started at January 2013. According to the Norwegian National Hospital Registry (http://www.NPR.no) approximately 400 patients sustaining a proximal humeral fracture are admitted in the including hospitals every year. At least 20% of these fractures are of a complex type according to the inclusion criterion, equaling 80 patients per year. We estimate that half of the patients will be eligible to inclusion; that is 40 patients/year. Inclusion of 120 patients should be completed during spring 2016.

### Interventions

Patients will be randomized to two groups, each of 60 patients:

Group 1 Sixty patients in the first group (intervention group) will be treated with a reversed total shoulder artroplasty (RTSA) of Delta type.

Group 2 Sixty patients in the second group (control group) will be operated with open reduction and internal fixation using a Philos plate and suture cerclages.

All patients will be enrolled in the study within 72 hours of hospital admission. Surgery will take place within 7 days thereafter.

### Description of treatment groups

#### Operative technique

Surgeons trained and experienced in the surgical technique before performing surgery on study participants, will perform all operations as a daytime procedure The surgeons skills and number of procedures from each center will be reported according to the criterias given by the Consort group.

1. Open reduction and internal fixation (Philos plate)
  The goal of surgery is anatomical reduction of the fracture and fracture stabilization to allow early mobilization. Surgery is performed under general anaesthesia with the patient in a beach-chair position and fluoroscopic control. The standardized approach is the deltopectoral, and will be preferred to a lateral deltoid split. An atraumatic reduction with respect to vascularization of bone fragments will be emphasized [50]. The osteofixation will be performed with an angular stable locking plate (Philos, Synthes®, Switzerland). After identification of the tubercle fragments, three size No5 braided polyester sutures are introduced around the insertion of the supraspinatus, infraspinatus and subscapularis tendons. The humeral head fragment(s) will be adjusted to anatomical inclination and retroversion using two K-wires as “joy-sticks” and temporarily transfixed by K-wires. The superior part of the locking plate is placed no higher than 1 cm below the top of the greater tubercle, and lateral to the bicipital groove to avoid interference with the anterior branch of the humeral circumflex artery. Also support of the medial part of the surgical neck by the most inferior locking screws according to Kralinger will be paid attention [51]. If necessary, a small buttress plate will be used medially to enhance the fixation (Figure 1). To avoid perforation of the tip of locking screws into the joint space during healing of the fracture due to “sinking” of the humeral head fragment for intracapsular C-type fractures, the screw tip should have a “clear space” of 8–10 mm to the subchondral bone. The need for bone grafting from the ipsilateral iliac crest or a bone substitute to enhanche stability after desimpacting the humeral head in a valgus-depressed fracture will be judged during the surgical procedure. Fluoroscopic control of fracture alignment and implant positioning is performed throughout the procedure.

**Figure 1:**
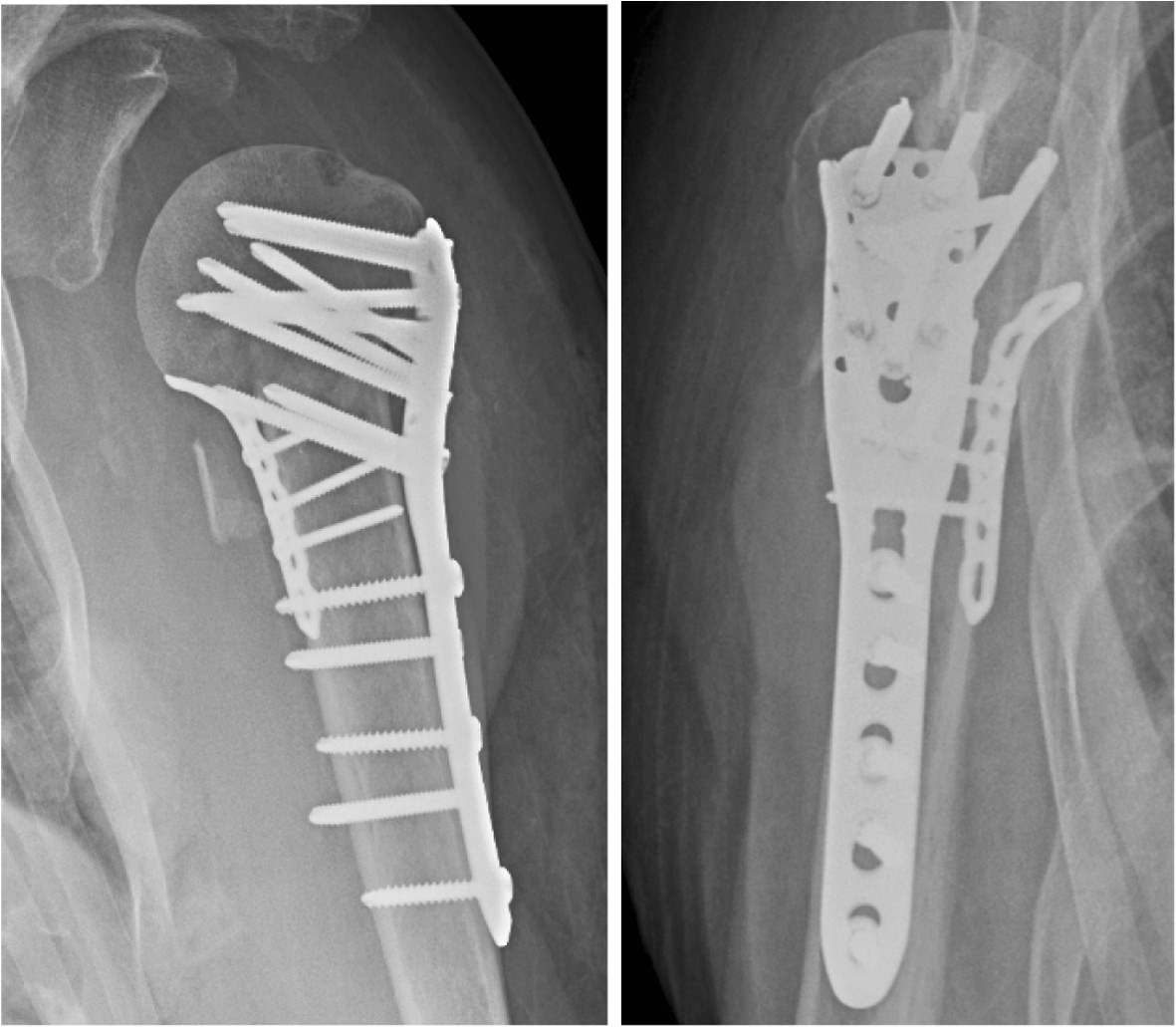
**Displaced proximal humeral fracture.** Control group. Angular stable implant (Philos) for osteofixation of a B2 varus fracture. 2.4 mm plate (Compact Foot, Synthez) has been used to secure the most frequent failure: Varus re-displacement.
2. Reversed Total Shoulder Arthroplasty (Delta-Xtend prosthesis® from DePuy)
  The goals to be met are restoring proper biomechanics, achieving an optimal range of motion and minimize patient discomfort. The standardized approach is the delto-pectoral, preferred to the supero-lateral approach to prevent any damage of the deltoid muscle [75]. A cemented monobloc humeral stem will be implanted. An important point to adress is how to decide the correct height of the humeral stem in the fractured proximal humerus, thus establishing a proper tension and stability of the prosthesis to avoid complications as instability [76]. The perioperative judgment of stability using the trial-implants are therefore regarded important. Furthermore, the fixation of the greater and lesser tubercles is crucial, as optimal recovery after RTSA partly depend on reattachment of the tubercles [39, 77]. Braided polyester suture -cerclages (no 5) engaging the insertion of the subscapular and infraspinatus tendons will secure the tubercles using three 2 mm drill holes in the metaphyseal part of the shaft in addition to bone graft from the humeral head. If the surgical neck fracture extends further distal than the humerus metaphysis, a diaphyseal wire-cerclage will be applied to prevent further diaphyseal fracturing. Finally, prevention of the well known complication “scapular notching” is important, and the largest 42 mm glenosphere will be used to help creating an inferior prosthetic overhang against the scapular neck [62] (Figure 2).

**Figure 2:**
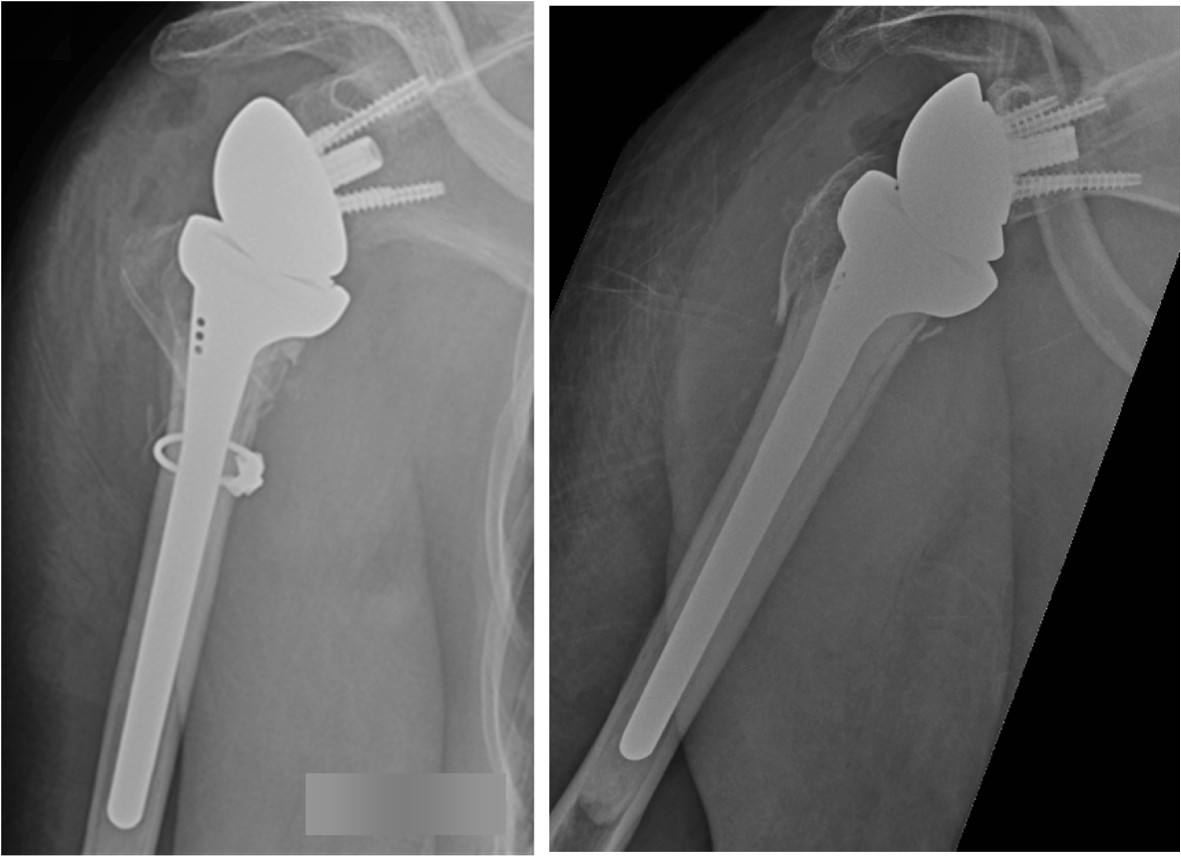
**Displaced proximal humeral fracture: Intervention group: Delta prothesis with overhang of the inferior part of the glenosphere component and wire-cerclage to control concommittant metaphyseal fractures.**

### Postoperative rehabilitation and physiotherapy

#### Education prior to inclusion of patients

Physiotherapists and orthopaedic surgeons from all centers attended a short educational program before patient inclusion, consisting of a lectures as well as work-shops for all attending physiotherapists.

After surgery the operated upper extremity will be immobilized in a sling bandage. Both patients treated with Philos plate and with RTSA will start self exercises and supported physiotherapy during the first three postoperative days.

All patients will receive instructions regarding daily home exercises. During the period of immobilization, patients will be instructed to perform antioedema exercises of all joints distal to the shoulder. By discharge from the hospital, they will be taught a standardized self-exercise program to start immediately after the immobilization period.

Outclinic physiotherapists will be notified by the hospital physiotherapist to ensure that rehabilitation starts immediately after removal of the sling. All outclinic physiotherapists will receive guidelines for rehabilitation and physiotherapy of the trial patients. The patients are discharged from physiotherapy treatment when shoulder function is considered to be satisfactory or any further progression seems unlikely.

#### The physiotherapy training protocol

The physiotherapy guideline is mainly equal for the two groups. There is a difference in the timeline during the six first weeks after the surgery due to the fact that while a fracture operated with an angular stable plate should be regarded as stable for immediate training, a RTSA cannot be allowed rotation and resistance exercises for some period to allow healing of the tubercles. The main elements of physical therapy are outlined in Table 1.

For patients in the intervention group operated with reverse shoulder arthroplasty, the importance of healing of the tubercles without displacement is emphazised [37, 39]. Therefore these patients are given physical therapy with assisted exercises from the first to the the sixth week. Activating the deltoid muscle with assisted physiotherapy may be equally important, as this has to control and stabilize the prosthesis.

For patients treated with open reduction and osteofixation with the Philos plate and threaded cerclages, the proximal humerus is regarded stable for exercises immediately after operation with limitations against resistance exercises during the first 6 weeks after surgery.

Information to all included patients and attending health workers are electronically available at http://www.oslo-universitetssykehus.no/aktuelt_/prosjekter_/Sider/brudd-i-overarmsbenet.aspx.

### Method of randomization

Randomization is performed by the NTNU web-CRF solution with separate blocks and stratification to age and gender for each collaborating hospital.

(https://webcrf.medisin.ntnu.no/client/index.php). Randomization of a new patient in one of the participating hospitals will generate a coded message to the project group with information of treatment and inclusion number for this hospital.

### Power of the trial and sample size

To calculate the sample size we used the primary outcome Constant Shoulder Score with a range from zero to 100 points. Using the mean values from the injured shoulder in a similar population after proximal humeral fractures, SD equals18 according to clinical experience [16, 43]. There are two groups to be compared: Intervention group and control group. The minimal clinically important difference has been decided to equal 10 points. Level of significance (α) equals 0.05. Given a power of 0.80 (β) the number required in each group is 51 patients. Due to a predicted loss of included patients during follow-up, we aim to include 60 patients in each group.

### Statistics

Statistical analyses will be performed with the SPSS 17.0 software or later version (SPSS Inc. Chicago, Illinois, USA). All outcomes will be reported as mean values and 95% confidence interval (95% CI) for both treatment groups and for each hospital separate, as well as for all hospitals together. In case of small data groups, bootstrapped 95% CI will be calcluated. Mean differences between the groups will be compared with independent *t*-test and Mann–Whitney test as appropriate at two and five years follow-up. Subgroup analyses for the two fracture groups B2 and C2 are planned to gain further information of effectiveness of treatment. Multiple imputation will be used to handle missing values. The QALY analysis from 15D outcome will be performed according to the specific 15D syntax for SPSS and completed with a one-way sensitivity analysis for each variable in the analysis.

The intention-to-treat principle will be adopted: Patients will be analysed according to their initial treatment group in case of cross over to the other group.

### Interim analyzes and stopping guidelines

Serious and unexpected adverse events will be reported according to recommendations given by the Consort Group. For the RTSA intervention group the primary focus will be postoperative infections, prosthesis dislocation, loosening of the glenosphere or the humeral stem and signs of scapular notching. Healing of the tubercles and signs of deltoid muscle dysfunction constitute secondary items. In the Philos group primary items will be postoperative infection and implant failure as pull-out and cut-out of the screws. The definition of infection is: a) Less serious infection: Superficial wound infection with sign of skin inflammation and/or a positive bacterial culture, without call for re-surgery. b) Serious infection: Any postoperative wound infection or sign of deep infection that call for re-surgery.

At 18 months an independent researcher from the main hospital OUS Orthopaedic Research Department will evaluate the complication rates and for the attending centers and correlate them to expected rates in the available literature. An unexpected high rate of complications in either group will be reported to the project group, deciding whether the randomization need to be closed.

### Trial registration

Trial has been reported on the http://www.ClinicalTrial.gov before inclusion started, and update will take place during the inclusion and follow-up if any serious unexpected events occur, and after completed inclusion of patients. ClinicalTrials.gov Identifier: NCT01737060.

### Consort statement

**Figure 3:**
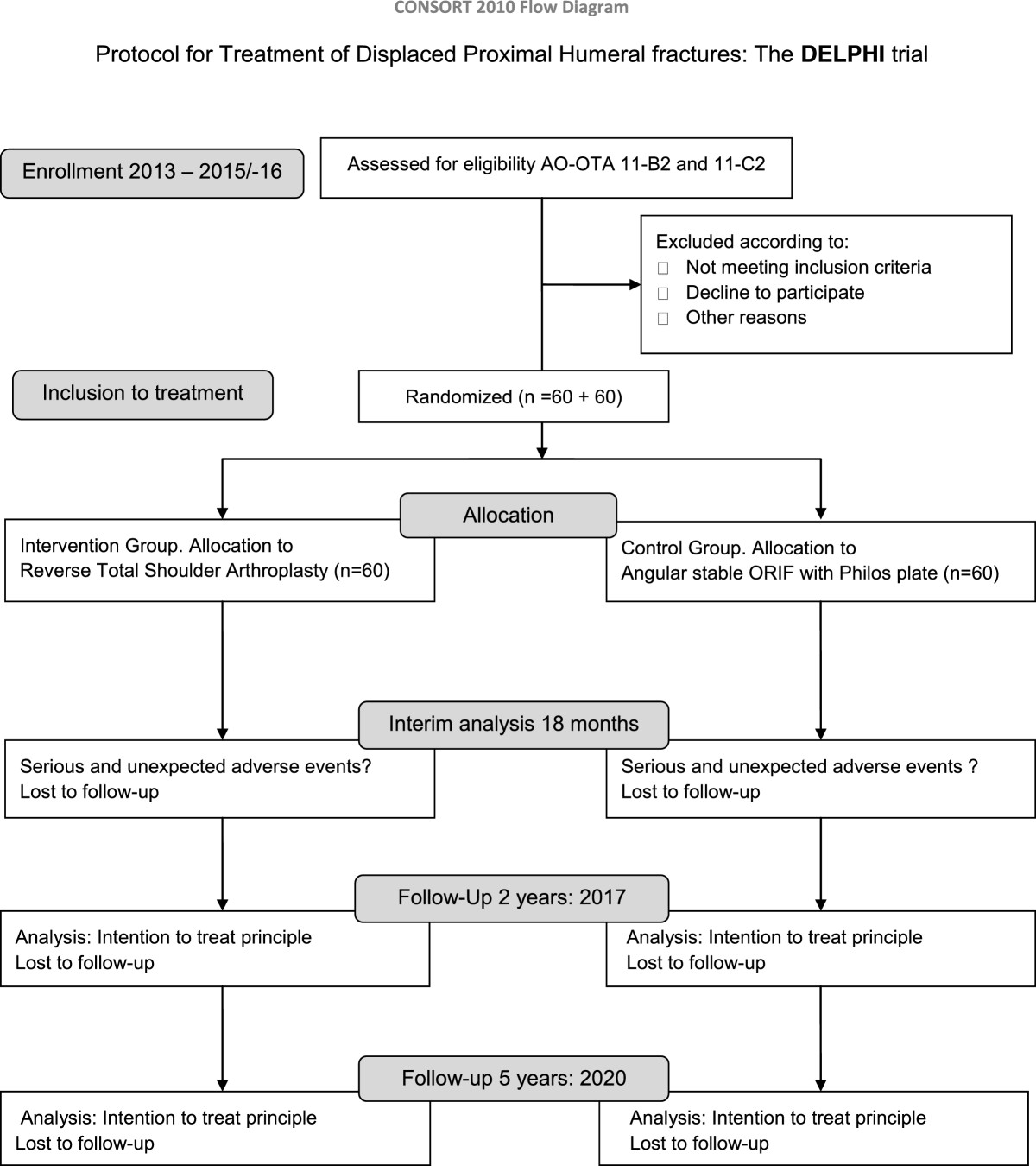
**Flow chart DELPHI- trial.** Timeline.

The study will be reported according to the Consort statement [4]. (http://www.consort-statement.org) A flow chart will be presented in line with the Consort recommendation Figure 3.

## Discussion

This protocol has been designed according to recommendations given by the Consort group [4]. The design of a randomized controlled study with a high level of evidence including more than 100 patients, with semi-blinded independent physical therapists collecting patient data gain important knowledge about proximal humeral fractures. A standardized surgical approach for both groups without compromising the deltoid muscle, reduces group differences, and a standarized educational program for all physiotherapists prior to examination of patients at follow-up contribute to a better methodological design and strengthen this study.

This study protocol has been adjusted according to our experience from former studies, both regarding outcomes, method of randomization and exclusion criterias [16, 17]. Also, to gain knowledge of the external validity of this study, all patients aged 65 to 85 years admitted to two of the recruiting hospitals will be grouped according to the exclusion criteria and AO classification, hopefully increasing our knowledge of the generalizability of our results. This generalizability and the question if the results of this trial will cause impact in the future treatment of displaced B2 and C2 proximal humeral fractures is a basic issue when a new trial is designed. Although it is more than 40 years since Charles Neer wrote his two important papers on classification and treatment of proximal humeral fractures, these papers are still frequently referred and the treatment is still under debate [1, 2]. The sparse number of studies at the highest level of evidence may be one of the reasons.

The trial may have potential weaknesses: Blinding of a trained physiotherapist examining a patient with a prosthetic replacement is difficult due to the mechanics of the implant. Another potential problem is that the primary outcome (Constant score) has a low reproducibility between different observers in a multicenter trial [67, 68]. However, the pre-trial training for all participating physiotherapists will reduce this bias.

Concerning the surgical treatment, not all parts of the treatment can be standardized, and may represent confounders. For the ORIF procedure the different fracture patterns, as well as the use of a medial calcar butress plate, may confound outcome. However, the surgeons perioperative individual judgement of the technically best treatment solution will have priority. All adjuvant treatment in addition to the Philos plate and thread cerclages around the tubercles will be reported.

Current recommendations in the litterature advise the use of reversed total shoulder arthroplasty in patients aged over 70 years [37]. Even so, the lower age limit in this trial has been set to 65 years. An acute proximal humeral fracture clearly represents a different problem compared with a planned revision of a failed arthroplasty or sequela after a malunited fracture. Kaplan-Meier survivorship analysis has showed acceptable outcomes for cuff tear arthropaties, while revision arthroplasties and fracture sequelae show less favourable results after 8–10 years [78, 79]. Primary joint replacement after fracture of the proximal humerus has gained better results than secondary revision arthroplasty, and hemi-arthroplasty give less predictable results than RTSA [39]. Thus, the youngest patients group in this trial may obtain a more predictable shoulder function for the next 8–10 years after a RTSA compared to using other prosthetic modalities. Therefore a potential decline in QoL will be delayed and possibly minimized, as older persons usually will have a natural decline in shoulder function and lower demand with higher age.

Some ongoing and recently completed trials deals with the issue of best treatment of proximal humeral fractures. Two recent randomized controlled trials of four-part humeral fractures treated either non-operatively or with hemiarthroplasty, could not show differences between the treatment groups at one year [80, 81]. Neither could our former trial comparing ORIF and non-operative treatment for three- and four part fractures prove any difference in favor of one of the treatments [16, 17].

However, no study has yet, to our knowledge, compared RTSA and ORIF. The only known trial comparing RTSA with another surgical option, is a small level 3 study, concluding that RTSA outperformed hemi-arthroplasty with regard to all outcomes measures assessed [57].

The RTSA principle has been developed during the last three decades [56, 82] reducing the problems of nonunion and/or resorption of the tubercles, a main problem flawing hemi-artroplasties [40]. Reducing the importance of the rotator cuff may, however, create increased load on the deltoid muscle and change the biomechanics of the glenohumeral joint. The potential problems with overload of the deltoid muscle and loosening of the glenosphere have been pointed out [38], and these problems certainly need further elucidation. Although several complications have been reported, the prostheses used in many of these studies are older RTSA designs, and the surgical indications mostly refer to patients going through revision surgery or rotator cuff defiencies [58]. In a short five years perspective RTSA may turn out to be a better and more predictable option than other prostetic replacements and ORIF, also in a health economic perspective [82]. However, in addition to this protocol including five years follow-up, we aim to follow these patients for ten years, to obtain information on long term RTSA survival after treatment of proximal humeral fractures.

## Collaborating Hospitals

Sykehuset i Telemark, Skien, Norway. Sykehuset Asker og Bærum, Vestre Viken, Norway. Sykehuset i Vestfold, Tønsberg, Norway. Sykehuset i Østfold, Fredrikstad, Norway. Sykehuset Førde, Førde, Norway. Diakonhjemmets sykehus, Oslo, Norway.

## Project group

Tore Fjalestad MD, PhD, Consultant, Head of project group. Jan Erik Madsen, Professor PhD, Head of Trauma, Orthopaedic Dept. Tom C. Ludvigsen MD, PhD, Head of Arthroscopy/Arthroplasty Upper Extremity. Petter Iversen MD, Consultant. Tone Wagle PT, Project researcher.

## Electronic supplementary material

Additional file 1:15D Secondary outcomes.(DOC 1 MB)

Below are the links to the authors’ original submitted files for images.

Authors’ original file for figure 1

Authors’ original file for figure 2

Authors’ original file for figure 3

## Abbreviations

RTSA: Reversed Total Shoulder Arthoplasty
Philos: Proximal Humeral Internal Locking System
DELPHI: The Delta-Philos trial
HA: Hemi Arthroplasty
AO-OTA: Arbeitsgruppe für Osteosynthesefragen - Orthopaedic Trauma Association
CSD: Constant Score Difference
OSS: Oxford Shoulder Score
15D: 15 Dimensional Helath Related Quality Of Life form (Harri Sintonen)
HQoL: Helath Related Quality Of Life
QALY: Quality Asjusted Life Years
ROM: Range Of Movement
MCID: Minimal Clinically Important Difference.

## References

[1] Neer CS (1970). Displaced proximal humeral fractures. I. Classification and evaluations. J Bone Joint Surg Am.

[2] Neer CS (1970). Displaced proximal humeral fractures. II. Treatment of three-part and four-part displacement. J Bone Joint Surg Am.

[3] Handoll HHG, Ollivere BJ (2010). Interventions for Treating Proximal Humeral Fractures in Adults. Cochrane Database of Systematic Reviews.

[4] Moher D, Schulz KF, Altmann DG (2001). The CONSORT statement: revised recommendations for improving the quality of reports of parallel- group randomised trials. Lancet.

[5] Court Brown CM, McQueen MM (2002). The relationship between fractures and increasing age with reference to the proximal humerus. Curr Orthop.

[6] Palvanen M, Kannus P, Niemi S, Parkkari J (2006). Update in the epidemiology of proximal humeral fractures. Clin Orthop Relat Res.

[7] Court-Brown CM, Garg A, McQueen MM (2001). The epidemiology of proximal humeral fractures. Acta Orthop Scand.

[8] Bengner U, Johnell O, Redlund-Johnell I (1988). Changes in the incidence of fracture of the upper end of the humerus during a 30 year period: a study of 2125 fractures. Clin Orthop.

[9] Palvanen MP, Kannus J, Parkkari T, Pitkäjarvi M, Pasanen I, Vuori I, Järvinen M (2000). The injury mechanisms of osteoporotic upper extremity fractures among older adults: a controlled study of 287 consecutive patients and their 108 controls. Osteoporosis Int.

[10] Kannus P, Palvanen M, Niemi S, Pakkari J, Järvinen M, Vuori I (2000). Osteoporotic fractures of the proximal humerus in elderly Finnish persons. Acta Orthop Scand.

[11] Kanis JA, Johnell O, Oden A, Sembo I, Redlund-Johnell I, Dawson A, De Laet C, Jonsson B (2000). Long-term risk of osteoporotic fracture in Malmø. Osteoporos Int.

[12] Giannoudis P, Tzioupis C, Almalki T (2007). Fracture healing in osteoporotic fractures: Is it really different? A basic science perspective. Injury.

[13] Fink HA, Ensrud KE, Nelson DB, Kerani RP, Schreiner PJ, Zhao Y, Cummings SR, Nevitt MC (2003). Disability after clinical fracture in postmenopausal women with low bone density: the fracture intervention trial (FIT). Osteoporos Int.

[14] Hodgson S (2006). Proximal humerus fracture rehabilitation. Clin Orthop Relat Res.

[15] Wright JG, Swiontkowski MF, Heckman JD (2003). Introducing levels of evidence to the journal. J Bone Joint Surg Am.

[16] Fjalestad T, Hole MØ, Hovden IA, Blücher J, Strømsøe K (2012). Surgical treatment with an angular stable plate for complex displaced proximal humeral fractures in elderly patients: a randomized controlled trial. J Orthop Trauma.

[17] Fjalestad T, Hole MØ, Jørgensen JJ, Strømsøe K, Kristiansen IS (2010). Health and cost consequences of surgical versus conservative treatment for a comminuted proximal humeral fracture in elderly patients. Injury.

[18] Bell JE, Leung BC, Spratt KF, Koval KJ, Weinstein JD, Goodman DC, Tosteson AN (2011). Trends and variation in incidence, surgical treatment, and repeat surgery of proximal humeral fractures in the elderly. J Bone Joint Surg Am.

[19] Marsh JL, Slongo TF, Angel J, Broderick JS, Creevey W, DeCoster TA, Prokuski L, Sirkin MS, Ziran B, Henley B, Audigè L (2007). Fracture and dislocation classification compendium - 2007: orthopaedic trauma association classification, database and outcomes committee. J Orthop Trauma.

[20] Hertel R, Hempfing A, Stiehler M, Leunig M (2004). Predictors of humeral head ischemia after intracapsular fracture of the proximal humerus. J Shoulder Elbow Surg.

[21] Nho SJ, Brophy RH, Barker JU, Cornell CN, MacGillivray JD (2007). Management of proximal humeral fractures based on current litterature. J Bone Joint Surg Am.

[22] Badman B, Frankle M, Keating C, Henderson L, Brooks J, Mighell M (2011). Results of proximal humeral locked plating with supplemental suture fixation of rotator cuff. J Shoulder Elbow Surg.

[23] Solberg BD, Moon CN, Franco DP, Paiement GD (2009). Surgical treatment of three and four-part proximal humeral fractures. J Bone Joint Surg Am.

[24] von Rüden C, Trapp O, Hierholzer C, Prohaska S, Wurm S, Bühren V (2014). Intramedullary nailing vs. locking plate osteosynthesis in proximal humeral fractures: Long-term outcome. Unfallchirurg.

[25] Hatzidakis AM, Shevlin MJ, Fenton DL, Curran-Everett D, Nowinski RJ, Fehringer EV (2011). Angular-stable locked intramedullary nailing of two-part surgical neck fractures of the proximal part of the humerus. A multicenter retrospective observational study. J Bone Joint Surg Am.

[26] Bogner R, Hübner C, Matis N, Auffarth A, Lederer S, Resch H (2008). Minimally-invasive treatment of three- and four-part fractures of the proximal humerus in elderly patients. J Bone Joint Surg (Br).

[27] Harrison AK, Gruson KI, Zmistowski B, Keener J, Galatz L, Williams G, Parsons BO, Flatow EL (2012). Intermediate outcomes following percutaneous fixation of proximal humeral fractures. J Bone Joint Surg Am.

[28] Kralinger F, Schwaiger R, Wambacher M, Farell E, Menth-Chiari W, Lajtai G, Hübner C, Resch H (2004). Outcome after primary hemiarthroplasty for fracture of the head of the humerus. J Bone Joint Surg (Br).

[29] Resch H, Povacz P, Frolich R, Wambacher M (1997). Percutaneous fixation of tree- and four-part fractures of the proximal humerus. J Bone Joint Surg (Br).

[30] Stableforth PG (1984). Four-part fractures of the neck of the humerus. J Bone Joint Surg Br Vol.

[31] Robinson CM, Page RS, Hill RMF, Sanders DL, Court-Brown CM, Wakefield AE (2003). Primary hemiartroplasty for treatment of proximal humeral fractures. J Bone Joint Surg Am.

[32] Bjørkenheim JM, Pajarinen J, Savolainen V (2004). Internal fixation of proximal humeral fractures with a locking compression plate. A retrospective evaluation of 72 patients followed for a minimum of 1 year. Acta Orthop Scand.

[33] Konrad G, Hirschmüller A, Audige L, Lambert S, Hertel R, Südkamp NP (2012). Comparison of two different locking plates for two-, three- and four-part proximal humeral fractures–results of an international multicentre study. Int Orthop.

[34] Gupta AK, Harris JD, Erickson BJ, Abrams GD, Bruce B, McCormick F, Nicholson GP, Romeo AA (2014). Surgical Management of Complex Proximal Humerus Fractures –A Systematic Review of 92 studies including 4,500 Patients. J Orthop Trauma.

[35] Olerud P, Ahrengart L, Ponzer S, Saving J, Tidermark J (2011). Internal fixation versus nonoperative treatment of displaced 3-part proximal humeral fractures in elderly patients: a randomized controlled trial. J Shoulder Elbow Surg.

[36] Misra A, Kapur R, Maffulli N (2001). Complex proximal humeral fractures in adults–a systematic review of management. Injury.

[37] Gallinet D, Clappas P, Garbuio P, Tropet Y, Obert L (2009). Three or four parts complex proximal humerus fractures: hemiarthroplasty versus reverse prosthesis: a comparative study of 40 cases. Orthop Traumatol Surg Res.

[38] Greiner SH, Diederichs G, Kröning I, Scheibel M, Perka CJ (2009). Tuberosity position correlates with fatty infiltration of the rotator cuff after hemiarthroplasty for proximal humeral fractures. Shoulder Elbow Surg.

[39] Sirveaux F, Roche O, Molé D (2010). Shoulder arthroplasty for acute proximal humerus fracture. Orthop Traumatol Surg Res.

[40] Nijs S, Broos P (2009). Outcome of shoulder hemiarthroplasty in acute proximal humeral fractures: a frustrating meta-analysis experience. Acta Orthop Belg.

[41] Boileau P, Krishnan SG, Tinsi L, Walch G, Coste JS, Molé D (2002). Tuberosity malposition and migration: reasons for poor outcomes after hemiarthroplasty for displaced fractures of the proximal humerus. J Shoulder Elbow Surg.

[42] Gallo RA, Sciulli R, Daffner RH, Altman DT, Altman GT (2007). Defining the relationship between rotator cuff injury and proximal humerus fractures. Clin Orthop Relat Res.

[43] Fjalestad T, Hole MØ, Blücher J, Hovden IA, Stiris MG, Strømsøe K (2010). Rotator cuff tears in proximal humeral fractures: an MRI cohort study in 76 patients. Arch Orthop Trauma Surg.

[44] Sommer C, Gautier E, Müller M, Helfet DL, Wagner M (2003). First clinical results of the Locking Compression Plate (LCP). Injury.

[45] Hertel R (2005). Fractures of the proximal humerus in osteoporotic bone. Osteoporos Int.

[46] Frangen TM, Müller EJ, Dudda M, Arens S, Muhr G, Kälicke T (2007). Proximal humeral fractures in geriatric patients. Is the angle-stable plate osteosynthesis really a breakthrough?. Acta Orthop Belg.

[47] Clavert P, Adam P, Bevort A, Bonnomet F, Kempf JF (2010). Pitfalls and complications with locking plate for proximal humerus fracture. J Shoulder Elbow Surg.

[48] Owsley KC, Gorczyca JT (2008). Fracture displacement and screw cutout after open reduction and locked plate fixation of proximal humeral fractures. J Bone Joint Surg Am.

[49] Agudelo J, Schürmann M, Stahel P, Helwig P, Morgan SJ, Zechel W, Bahrs C, Parekh A, Ziran B, Williamns A, Smith W (2007). Analysis of efficacy and failure in proximal humerus fractures treated with locking plates. J Orthop Trauma.

[50] Robinson CM, Amin AK, Godley KC, Murray IR, White TO (2011). Modern perspectives of open reduction and plate fixation of proximal humerus fractures. J Orthop Trauma.

[51] Kralinger F, Unger S, Wambacher M, Smekal V, Schmoelz W (2009). The medial periosteal hinge, a key structure in fractures of the proximal humerus: a biomechanical cadaver study of its mechanical properties. J Bone Joint Surg (Br).

[52] Bahrs C, Rolauffs B, Stuby F, Dietz K, Weise K, Helwig P (2010). Effect of proximal humeral fractures on the age-specific prevalence of rotator cuff tears. J Trauma.

[53] Wilmanns C, Bonnaire F (2002). Rotator cuff alterations resulting from humeral head fractures. Injury.

[54] Speck M, Regazzoni P (1997). Four fragment fractures of the proximal humerus. Alternative strategies for surgical treatment. Unfallchirurg.

[55] Becker R, Pap G, Machner A, Neumann WH (2002). Strength and motion after hemiarthroplasty in displaced four-fragment fracture of the proximal humerus: 27 patients followed for 1–6 years. Acta Orthop Scand.

[56] Cazeneuve JF, Cristofari DJ (2006). Grammont reversed prosthesis for acute complex fracture of the proximal humerus in an elderly population with 5 to 12 years follow-up. Rev Chir Orthop Reparatrice Appar Mot.

[57] Garrigues GE, Johnston PS, Pepe MD, Tucker BS, Ramsey ML, Austin LS (2012). Hemiartroplasty versus reverse total shoulder arthroplasty for acute proximal humeral fractures in elderly patients. Orthopedics.

[58] Mahmood A, Malal JJ, Waseem M (2013). Reverse shoulder arthroplasty – a litterature review. Open Orthop J.

[59] Reitman RD, Kerzhner E (2011). Reverse shoulder arthoplasty as treatment for comminuted proximal humeral fractures in elderly patients. Am J Orthop (Belle Mead NJ).

[60] Moosmeyer S, Smith HJ, Tariq R, Larmo A (2009). Prevalence and characteristics of asymptomatic tears of the rotator cuff: an ultrasonographic and clinical study. J Bone Joint Surg (Br).

[61] Cazeneuve JF, Cristofari DJ (2009). Delta III reverse shoulder arthroplasty: radiological outcome for acute complex fractures of the proximal humerus in elderly patients. Orthop Traumatol Surg Res.

[62] de Wilde LF, Poncet D, Middermacht B, Ekelund A (2010). Prosthetic overhang is the most effective way to prevent scapular conflict in a reverse total shoulder prosthesis. Acta Orthop.

[63] Sintonen H (2001). The 15D instrument of health-related quality of life: properties and applications. Ann Med.

[64] Dawson J, Rogers K, Fitzpatric R, Carr A (2009). The Oxford shoulder score revisited. Arch Orthop Trauma Surg.

[65] Constant CR, Murley AHG (1987). A clinical method of functional assessment of the shoulder. Clin Othop Rel Resarch.

[66] Constant CR, Gerber C, Emery RJ (2008). A review of the Constant score: modifications and guidelines for its use. J Shoulder Elbow Surg.

[67] Conboy VB, Morris RW, Kiss J, Carr AJ (1996). An evaluation of the constant - murley shoulder assessment. J Bone Joint Surg (Br).

[68] Kukkonen J, Kauko T, Vahlberg T, Joukainen A, Aärimaa V (2013). Investigating minimal clinically important difference for Constant score in patients undergoing rotator cuff surgery. J Shoulder Elbow Surg.

[69] Harvie P, Pollard TCB, Chennagiri RJ, Carr AJ (2005). The use of outcome scores in surgery of the shoulder. J Bone Joint Surg (Br).

[70] Edelson G, Saffuri H, Obid E, Vigder F (2009). The three-dimensional anatomy of proximal humeral fractures. J Shoulder Elbow Surg.

[71] Brunner A, Honingmann P, Treumann T, Babst R (2009). The impact of stereo-visualisation of three-dimensional CT datasets on inter- and intraobserver reliability of the AO/OTA and Neer classifications in the assessment of fractures of the proximal humerus. J Bone Joint Surg (Br).

[72] Sadoghi P, Leithner A, Vavken P, Hölzer A, Hochreiter J, Weber G, Pietschmann MF, Müller PE (2011). Infraglenoidal scapular notching in reverse total shoulder replacement: a prospective series of 60 cases and systematic review of the literature. BMC Musculoskelet Disord.

[73] Levigne C, Boileau P, Favard L, Garaud P, Mole D, Sirveaux F, Walch G (2008). Scapular notching in reverse shoulder arthroplasty. J Shoulder Elbow Surg.

[74] Lévigne C, Garret J, Boileau P, Alami G, Favard L, Walch G (2011). Scapular notching in reverse shoulder arthroplasty: is it important to avoid it and how?. Clin Orthop Relat Res.

[75] Lädermann A, Lubbeke A, Collin P, Edwards TB, Sirveaux F, Walch G (2011). Influence of surgical approach on functional outcome in reverse shoulder arthroplasty. Orthop Traumatol Surg Res.

[76] Smith CD, Guyver P, Bunker TD (2012). Indications for reverse shoulder replacement: a systematic review. J Bone Joint Surg (Br).

[77] Levy J, Badman B (2011). Reverse shoulder prosthesis for acute four-part fracture. tuberosity fixation using a horseshoe graft. J Orthop Trauma.

[78] Wellmann M, Struck M, Pastor MF, Gettmann A, Windhagen H, Smith T (2013). Short and midterm results of reverse shoulder arthroplasty according to the preoperative etiology. Arch Orthop Trauma Surg.

[79] Ianotti J, Madden M (2012). Long Term Results for RTSA Cleveland Clinic. Abstract 8.th Biennial AAOS/ASES/Shoulder and Elbow.

[80] Boons HW, Goosen JH, van Grinsven S, van Susante JL, van Loon CJ (2012). Hemiartroplasty for humeral four-part fractures for patients 65 years and older: a randomized controlled tria. Clin Othop Relat Res.

[81] Olerud P, Ahrengart L, Ponzer S, Saving J, Tidermark J (2011). Hemiarthroplasty versus nonoperative treatment of displaced 4-part proximal humeral fractures in elderly patients: a randomized controlled trial. J Shoulder Elbow Surg.

[82] Chalmers PN, Slikker W, Mall NA, Gupta AK, Rahman Z, Enriquez D, Nicholson GP (2013). Reverse total shoulder arthroplasty for acute proximal humeral fracture: comparison to open reduction-internal fixation and hemiarthroplasty. J Shoulder Elbow Surg.

[CR83] The pre-publication history for this paper can be accessed here:http://www.biomedcentral.com/1471-2474/15/323/prepub

