# Supplementary material for: Clinical investigation for displaced proximal humeral fractures in the elderly: a randomized study of two surgical treatments: reverse total prosthetic replacement versus angular stable plate Philos (The DELPHI-trial)
Source: BMC Musculoskelet Disord. 2014 Sep 28;15:323. doi: 10.1186/1471-2474-15-323 (PMC4247153; doi:10.1186/1471-2474-15-323)
Supplement: Supplementary file 1 — Additional file 1:15D Secondary outcomes.(DOC 1 MB) [file 12891_2013_2320_MOESM1_ESM.doc]

**Additional file 1**

**Appendix A 15D Outcome**

**QUALITY OF LIFE QUESTIONNAIRE (15D©)** Harri Sintonen

15D English version From Harri Sintonen [www.15D-instrument.net](http://www.15D-instrument.net/).Modification: Question 15: “Sexual activity” is excluded. Questionnaire is validated for this adjustment.

**QUESTION 1. MOBILITY**

1 ( ) I am able to walk normally (without difficulty) indoors, outdoors and on stairs.

2 ( ) I am able to walk without difficulty indoors, but outdoors and/or on stairs I have slight difficulties.

3 ( ) I am able to walk without help indoors (with or without an appliance), but outdoors and/or on stairs only with considerable difficulty or with help from others.

4 ( ) I am able to walk indoors only with help from others.

5 ( ) I am completely bed-ridden and unable to move about.

**QUESTION 2. VISION**

1 ( ) I see normally, i.e. I can read newspapers and TV text without difficulty (with or without glasses).

2 ( ) I can read papers and/or TV text with slight difficulty (with or without glasses).

3 ( ) I can read papers and/or TV text with considerable difficulty (with or without glasses).

4 ( ) I cannot read papers or TV text either with glasses or without, but I can see enough to walk about without guidance.

5 ( ) I cannot see enough to walk about without a guide, i.e. I am almost or completely blind.

**QUESTION 3. HEARING**

1 ( ) I can hear normally, i.e. normal speech (with or without a hearing aid).

2 ( ) I hear normal speech with a little difficulty.

3 ( ) I hear normal speech with considerable difficulty; in conversation I need voices to be louder than normal.

4 ( ) I hear even loud voices poorly; I am almost deaf.

5 ( ) I am completely deaf.

**QUESTION 4. BREATHING**

1 ( ) I am able to breathe normally, i.e. with no shortness of breath or other breathing difficulty.

2 ( ) I have shortness of breath during heavy work or sports, or when walking briskly on flat ground or slightly uphill.

3 ( ) I have shortness of breath when walking on flat ground at the same speed as others my age.

4 ( ) I get shortness of breath even after light activity, e.g. washing or dressing myself.

5 ( ) I have breathing difficulties almost all the time, even when resting.

**QUESTION 5. SLEEPING**

1 ( ) I am able to sleep normally, i.e. I have no problems with sleeping.

2 ( ) I have slight problems with sleeping, e.g. difficulty in falling asleep, or sometimes waking at night.

3 ( ) I have moderate problems with sleeping, e.g. disturbed sleep, or feeling I have not slept enough.

4 ( ) I have great problems with sleeping, e.g. having to use sleeping pills often or routinely, or usually waking at night and/or too early in the morning.

5 ( ) I suffer severe sleeplessness, e.g. sleep is almost impossible even with full use of sleeping pills, or staying awake most of the night.

**QUESTION 6. EATING**

1 ( ) I am able to eat normally, i.e. with no help from others.

2 ( ) I am able to eat by myself with minor difficulty (e.g. slowly, clumsily, shakily, or with special appliances).

3 ( ) I need some help from another person in eating.

4 ( ) I am unable to eat by myself at all, so I must be fed by another person.

5 ( ) I am unable to eat at all, so I am fed either by tube or intravenously.

**QUESTION 7. SPEECH**

1 ( ) I am able to speak normally, i.e. clearly, audibly and fluently.

2 ( ) I have slight speech difficulties, e.g. occasional fumbling for words, mumbling, or changes of pitch.

3 ( ) I can make myself understood, but my speech is e.g. disjointed, faltering, stuttering or stammering.

4 ( ) Most people have great difficulty understanding my speech.

5 ( ) I can only make myself understood by gestures.

**QUESTION 8. ELIMINATION**

1 ( ) My bladder and bowel work normally and without problems.

2 ( ) I have slight problems with my bladder and/or bowel function, e.g. difficulties with urination, or loose or hard bowels.

3 ( ) I have marked problems with my bladder and/or bowel function, e.g. occasional 'accidents', or severe constipation or diarrhea.

4 ( ) I have serious problems with my bladder and/or bowel function, e.g. routine 'accidents', or need of catheterization or enemas.

5 ( ) I have no control over my bladder and/or bowel function.

**QUESTION 9. USUAL ACTIVITIES**

1 ( ) I am able to perform my usual activities (e.g. employment, studying, housework, free-time activities) without difficulty.

2 ( ) I am able to perform my usual activities slightly less effectively or with minor difficulty.

3 ( ) I am able to perform my usual activities much less effectively, with considerable difficulty, or not completely.

4 ( ) I can only manage a small proportion of my previously usual activities.

5 ( ) I am unable to manage any of my previously usual activities.

**QUESTION 10. MENTAL FUNCTION**

1 ( ) I am able to think clearly and logically, and my memory functions well

2 ( ) I have slight difficulties in thinking clearly and logically, or my memory sometimes fails me.

3 ( ) I have marked difficulties in thinking clearly and logically, or my memory is somewhat impaired.

4 ( ) I have great difficulties in thinking clearly and logically, or my memory is seriously impaired.

5 ( ) I am permanently confused and disoriented in place and time.

**QUESTION 11. DISCOMFORT AND SYMPTOMS**

1 ( ) I have no physical discomfort or symptoms, e.g. pain, ache, nausea, itching etc.

2 ( ) I have mild physical discomfort or symptoms, e.g. pain, ache, nausea, itching etc.

3 ( ) I have marked physical discomfort or symptoms, e.g. pain, ache, nausea, itching etc.

4 ( ) I have severe physical discomfort or symptoms, e.g. pain, ache, nausea, itching etc.

5 ( ) I have unbearable physical discomfort or symptoms, e.g. pain, ache, nausea, itching etc.

**QUESTION 12. DEPRESSION**

1 ( ) I do not feel at all sad, melancholic or depressed.

2 ( ) I feel slightly sad, melancholic or depressed.

3 ( ) I feel moderately sad, melancholic or depressed.

4 ( ) I feel very sad, melancholic or depressed.

5 ( ) I feel extremely sad, melancholic or depressed.

**QUESTION 13. DISTRESS**

1 ( ) I do not feel at all anxious, stressed or nervous.

2 ( ) I feel slightly anxious, stressed or nervous.

3 ( ) I feel moderately anxious, stressed or nervous.

4 ( ) I feel very anxious, stressed or nervous.

5 ( ) I feel extremely anxious, stressed or nervous.

**QUESTION 14. VITALITY**

1 ( ) I feel healthy and energetic.

2 ( ) I feel slightly weary, tired or feeble.

3 ( ) I feel moderately weary, tired or feeble.

4 ( ) I feel very weary, tired or feeble, almost exhausted.

5 ( ) I feel extremely weary, tired or feeble, totally exhausted.

Sintonen H. The 15D instrument of health-related quality of life: properties and applications. *Ann Med* 2001; 33: 328 - 336.

**Economic questionnaire** (English version)

EXPENSES PROXIMAL HUMERAL FRACTURES MØH, TF 2012

□ BEFORE □ AT 3 MONTHS □ AT 6 MONTHS □ AT 1 YEAR □ AT 2 / 5 YEAR

**1)** Time hospital stay? (Question at 3 months) Days ……………

**2)** Living place since last follow-up?

 At home: From……… Until ………….

 Hospital rehabilitation centre: From……… Until ………….

 Other rehabilitation centres: From……… Until ………….

 Nursing home (short stay): From……… Until ………….

**3 )** If stay at home, do You need home nursing  Yes  No

 Once a week  Twice a week  More

**4a)** Number of visits to a physiotherapist since last follow-up ?

 0  1-5  6-10  11-15  16-20  21-25

 26-30  31-35  36-40  41-45  46-50

 If more frequently, please estimate: …………

**4b)** Did You need help from family or relatives who had to stay away from their daily job?

 Yes  Always  Frequently  Seldom  Newer

**4c)** I travelled by:  I walked  Used my own car  Bus  Taxi

**5a)** How many times did You see Your General Practitioner since last follow-up?

Number of visits ……………

**5b)** Did You need help from family or relatives who had to stay away from their daily job?

 Yes  No

**5c)** I travelled by:  I walked  Used my own car  Bus  Taxi

**6a)** Did You have any hospital stay since last follow-up?  No  Yes (Days ……. )

6b) Reason for hospital stay:  No  Yes

If yes: Due to the shoulder injury?  Other reasons 

**7)** Have You had a sick-leave due to Your shoulder injury?  No  Yes (Days ……. )

**Appendix B**

**RADIOLOGIC SCORE**

| **Variable** | **RTSA** | **Philos** |
| --- | --- | --- |
| Inclination humeral head | - | Yes |
| Center of humeral head with reference to glenoid | - | Yes |
| Screw cutout humeral head – primary / secondary | - | Yes |
| Subacromial distance (RC) | - | Yes |
| Avascular head necrosis Score □ | - | Yes |
| Osteofix. failure | - | Yes |
| Non-union | - | Yes |
| Prostetic radiolucent line Stem / Globe | Yes | - |
| Greater tubercule position / height | Yes | Yes |
| Glenoshpere position | Yes | - |
| Glenoid notching | Yes | - |
| Ectopic bone formation | Yes | Yes |


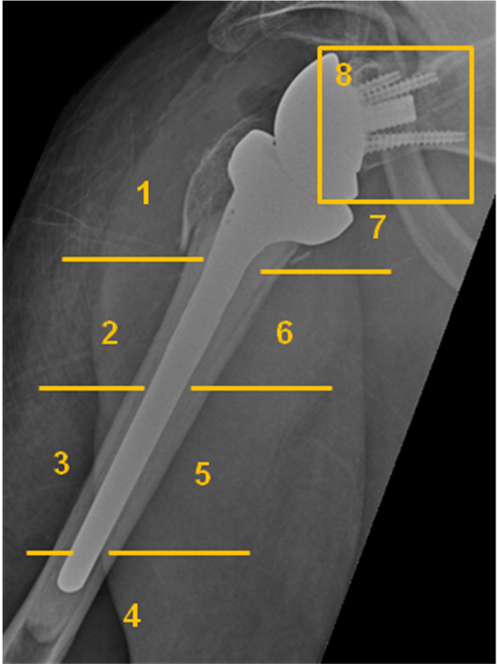


**Figure 3.**

Radiolucent lines will be measured in seven different zones around the humeral stem, and beneath the glenoid baseplate and around the central peg and fixation screws according to Lévigne et al. 2011.


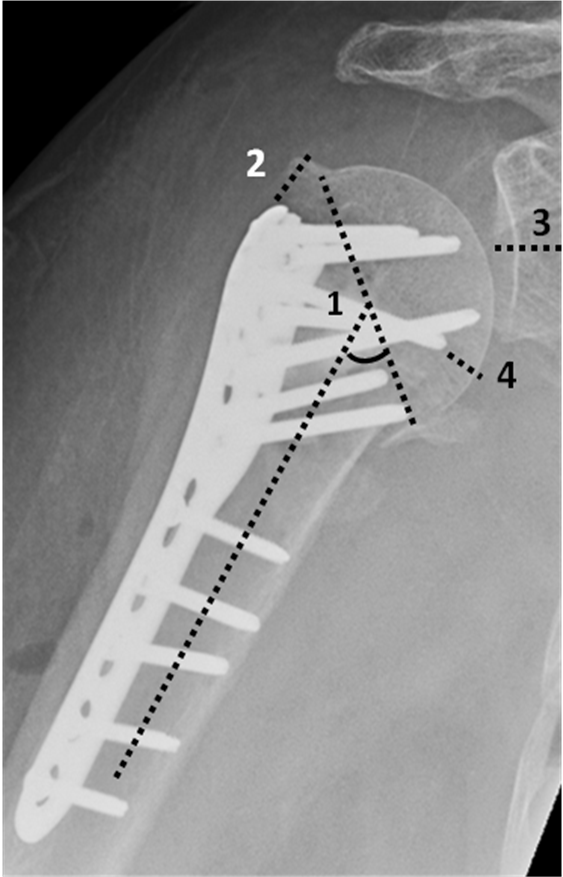


**Figure 4**

Radiographic evaluation Philos plate: Post-operative and follow-ups.

1. Humeral head inclination.

2. Position of greater tubercle. Plate height.

3. Alignment of the humeral head in glenoid fossa.

4. Distance screw tip - joint surface.


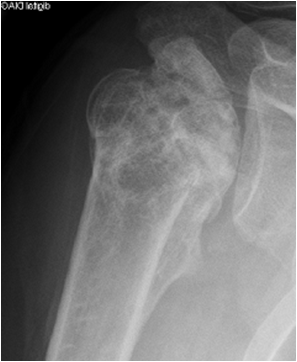


**Figure 5**

An example of scoring of advanced avascular humeral head necrosis.

Both trabecular changes in the humeral head >50% and segmental collapse equals score 0.
