# Supplementary figures and images for: Clinical investigation for displaced proximal humeral fractures in the elderly: a randomized study of two surgical treatments: reverse total prosthetic replacement versus angular stable plate Philos (The DELPHI-trial)
Source: BMC Musculoskelet Disord. 2014 Sep 28;15:323. doi: 10.1186/1471-2474-15-323 (PMC4247153; doi:10.1186/1471-2474-15-323)

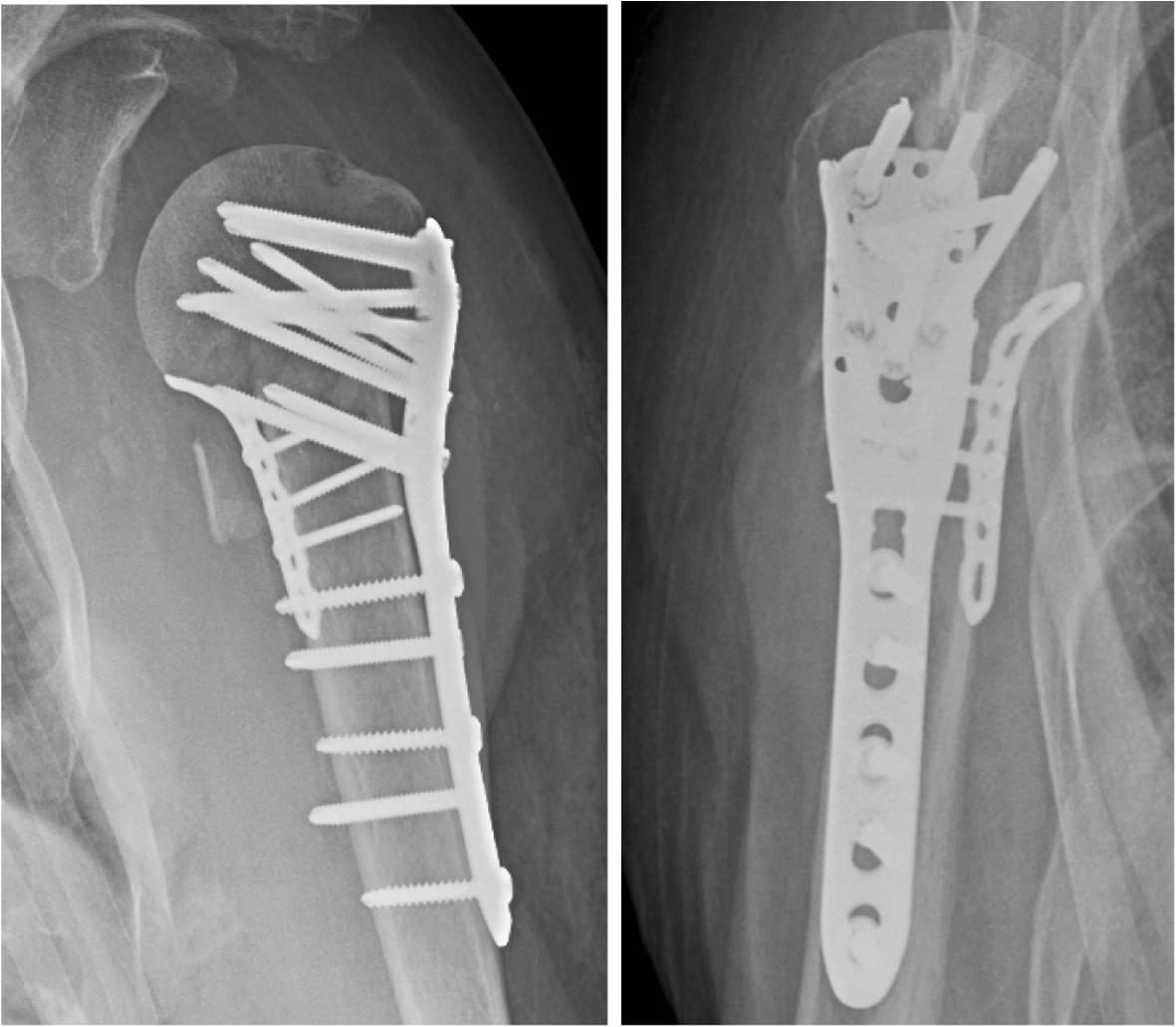

Supplement: Supplementary file 2 — Authors’ original file for figure 1 [file 12891_2013_2320_MOESM2_ESM.tif]

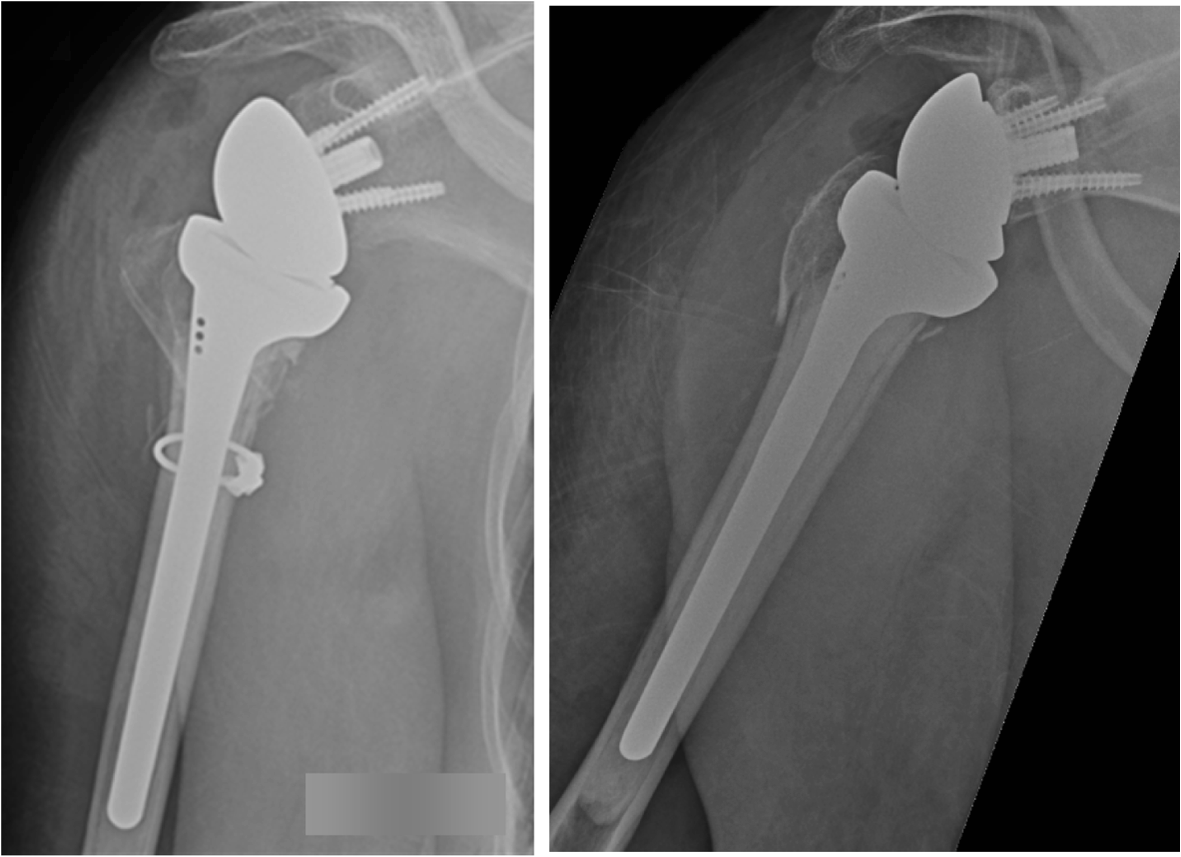

Supplement: Supplementary file 3 — Authors’ original file for figure 2 [file 12891_2013_2320_MOESM3_ESM.tif]

Protocol for Treatment of Displaced Proximal Humeral fractures: The **DELPHI** trial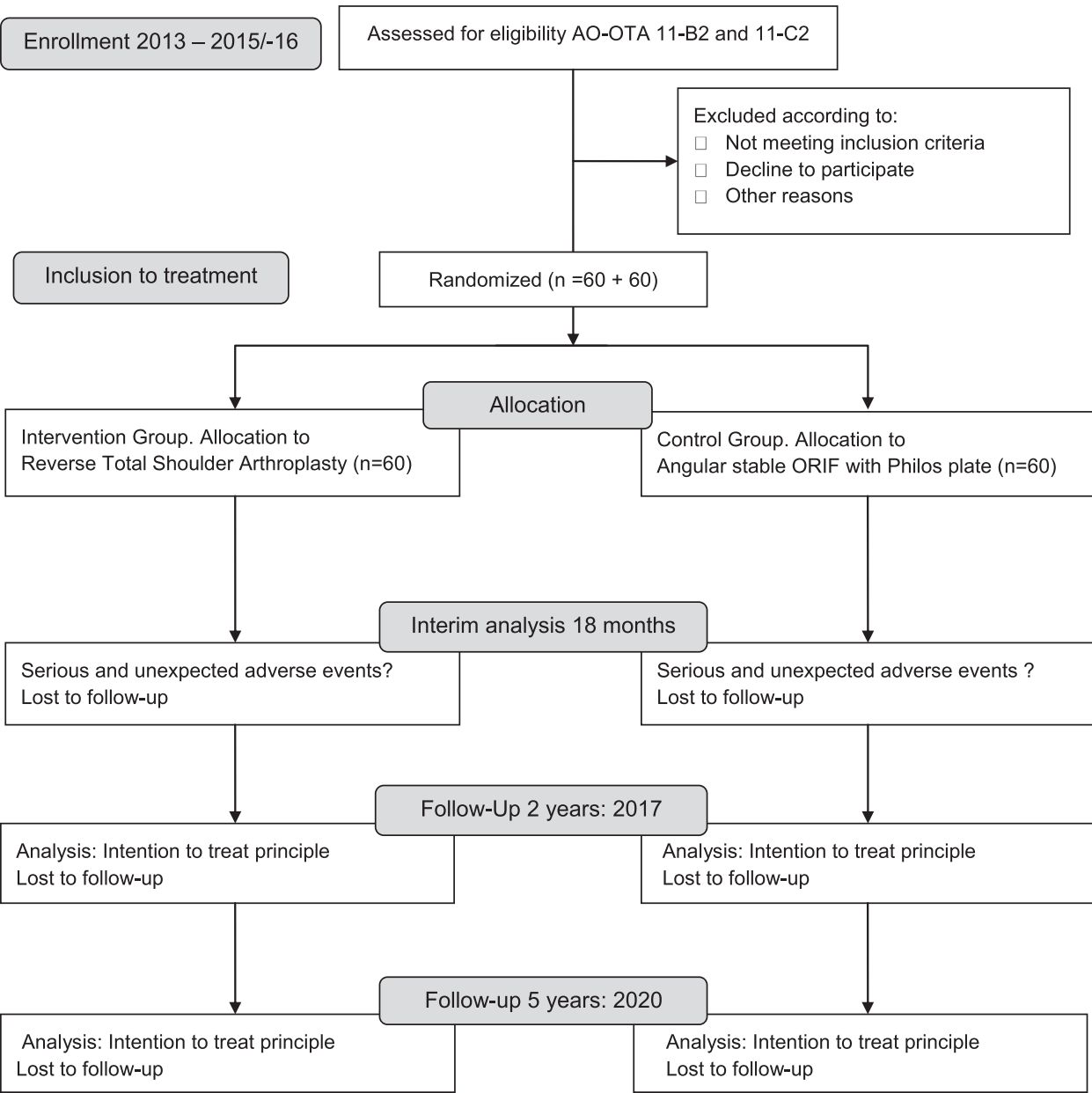

Supplement: Supplementary file 4 — Authors’ original file for figure 3 [file 12891_2013_2320_MOESM4_ESM.pdf]
